# Information Extraction of Doctoral Theses Using Two Different Large Language Models vs Health Services Researchers: Development and Usability Study

**DOI:** 10.2196/77707

**Published:** 2025-12-10

**Authors:** Jonas Cittadino, Pia Traulsen, Teresa Schmahl, Larisa Wewetzer, Julia Cummerow, Kristina Flägel, Christoph Strumann, Katja Goetz, Jost Steinhäuser

**Affiliations:** 1 Institute of Family Medicine, University Hospital Schleswig-Holstein, Maria-Goeppert-Straße 9a, Lübeck, 23538, Germany, 49 451 3101 ext 8001

**Keywords:** ChatGPT, family medicine, doctoral thesis, GPT-4o, Gemini, artificial intelligence, AI

## Abstract

**Background:**

The Archive of German-Language General Practice (ADAM) stores about 500 paper-based doctoral theses published from 1965 to today. Although they have been grouped in different categories, no deeper systematic process of information extraction (IE) has been performed yet. Recently developed large language models (LLMs) like ChatGPT have been attributed the potential to help in the IE of medical documents. However, there are concerns about LLM hallucinations. Furthermore, there have not been reports regarding their usage in nonrecent doctoral theses yet.

**Objective:**

The aim of this study is to analyze if LLMs can help to extract information from doctoral theses by using GPT-4o and Gemini-1.5-Flash for paper-based doctoral theses in ADAM.

**Methods:**

We randomly selected 10 doctoral theses published between 1965 and 2022. After preprocessing, we used two different LLM pipelines, using models by OpenAI and Google. Pipelines were used to extract dissertation characteristics and generate uniform abstracts. Furthermore, one pooled human-generated abstract was written for comparison. Furthermore, blinded raters were asked to evaluate LLM-generated abstracts in comparison to the human-generated ones. Bidirectional encoder representations from transformers scores were calculated as the evaluation metric.

**Results:**

Relevant dissertation characteristics and keywords could be extracted for all theses (n=10): institute name and location, thesis title, author name(s), and publication year. For all except one doctoral thesis, an abstract could be generated using GPT-4o, while Gemini-1.5-Flash provided abstracts in all cases (n=10). The modality of abstract generation showed no influence in raters’ evaluation using the nonparametric Kruskal-Wallis test for independent groups (*P*=.44). The creation of LLM-generated abstracts was estimated to be 24-36 times faster than creation by humans. Evaluation metrics showed moderate-to-high semantic similarity (mean bidirectional encoder representations from transformers *F*_1_-score, GPT-4o: 0.72 and Gemini: 0.71). Translation from German into English did not result in a loss of information (n=10).

**Conclusions:**

An accumulating body of unpublished doctoral theses makes it difficult to extract relevant evidence. Recent advances in LLMs like ChatGPT have raised expectations in text mining, but they have not yet been used in the IE of “historic” medical documents. This feasibility study suggests that both models (GPT-4o and Gemini-1.5-Flash) helped to accurately simplify and condense doctoral theses into relevant information, while LLM-generated abstracts were perceived as similar to human-generated ones, were semanticly similar, and took about 30 times less time to create. This pilot study demonstrates the feasibility of a regular office-scanning workflow and use of general-purpose LLMs to extract relevant information and produce accurate abstracts from ADAM doctoral theses. Taken together, this information could help researchers to better search the family medicine scientific literature over the last 60 years, helping to develop current research questions.

## Introduction

By their nature, archives contain a large amount of information. Thirty years ago, the German Society of General Practitioners and Family Physicians (DEGAM) began to centrally store all doctoral theses of the specialty. This collection is now part of the Archive of German-Language General Practice (ADAM) [1]. ADAM was primarily established to gather historical documents from the beginning of family medicine as a specialty in German-speaking countries (Germany, Austria, and Switzerland) and now stores a large number of documents of different types and formats, including many documents written with a typewriter [2]. Lately, there have been efforts to categorize 802 available theses [3,4]. During this process, one finding was that it is hard to find detailed information, especially for theses from before 2000. This may be because an estimated 50% of all dissertations are not published in a journal but rather are paper-based only [4]. Within ADAM, this is true for 553 dissertations. Therefore, as yet, there is no process for extracting relevant information, creating uniform abstracts, and making them available to the public and other researchers.

With the release of large language models (LLMs) such as ChatGPT by OpenAI and Gemini by Google, hopes have been raised regarding their use for medicine and medical documents [5]. The reduction of hallucination, the term for information made up by an LLM, is crucial for medical scenarios and has been the subject of a recent project [6]. Although previous work on LLMs has shown effectiveness in terms of information extraction (IE) from scientific texts [7,8], to our knowledge, there has not yet been a reported use of LLMs regarding IE from doctoral theses. Hence, the aim of this feasibility study was to analyze if LLMs and natural language processing can extract relevant information and generate uniform abstracts of doctoral theses from the field of family medicine in ADAM and how these artificial intelligence–generated abstracts are perceived by scientists.

## Methods

### Dissertation Characteristics

For this analysis, we randomly selected 10 dissertations from ADAM, ranging from the earliest dissertation (1965) to the most recent one (2022). All dissertations were in PDF format, either made available by the original author or generated by study personnel who scanned paper-based dissertations using a regular multifunctional office printer. When making our selection, we included different time periods of submission and both types of digitization in equal numbers. Therefore, we focused on dissertations from different time periods and different methods of digitization. Although the digitization of documents is a current fundamental question in archive research, only a few standards exist [9].

### Ethical Considerations

Since all selected dissertations were publicly accessible through ADAM, a formal ethics review was deemed unnecessary. However, informed consent was obtained from all copyright holders of the selected dissertations. According to our institutional practice (University Hospital Schleswig-Holstein), projects based solely on publicly available documents with author consent do not require review by the ethics committee. This analysis followed the transparent reporting of a multivariable model for individual prognosis or diagnosis (TRIPOD)+LLM guidelines (see Multimedia Appendix 1) [10].

### Analysis

For each PDF file, we created an analytical pipeline including the following steps: preprocessing, extracting information from the title page, creating a uniform abstract, and translating newly generated abstracts into English. We evaluated two LLMs in this study: GPT-4o [11] by OpenAI and Gemini-1.5-Flash [12] by Google.

#### GPT-4o

**Figure 1.**
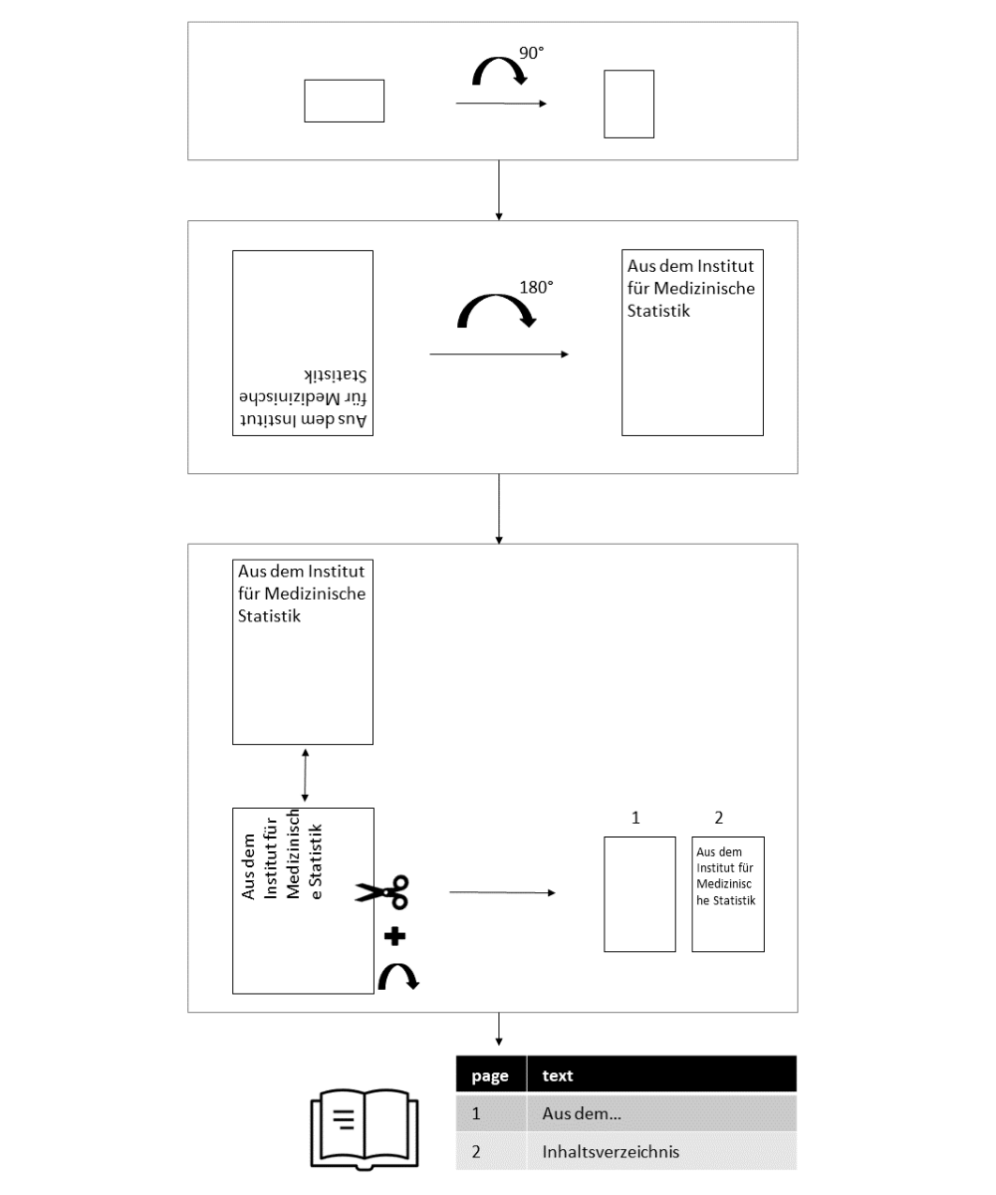
Preprocessing of scanned dissertation title pages prior to analysis. Workflow of preprocessing steps applied to scanned dissertation title pages from German medical faculties. The process included rotation correction, text segmentation, and optical character recognition to prepare documents for automated metadata extraction.

Preprocessing of all files in the GPT-4o pipeline was done by bringing all files into a vertical layout format before they were rotated and/or divided depending on text alignment (Figure 1). If the extracted text resulted in nonsense, we used optical character recognition (OCR) [13] for extraction of all pages. Then, the content of each page was stored as a string in a dictionary.

For further analysis, we used LLMs by OpenAI, namely text-davinci-003, GPT-3.5-turbo, and GPT-4o [11], using a temperature [14] of 0.2 to reduce hallucination. All models are pretrained and transformer-based, meaning that the model supervised its learning itself and understands context [15].

**Figure 2.**
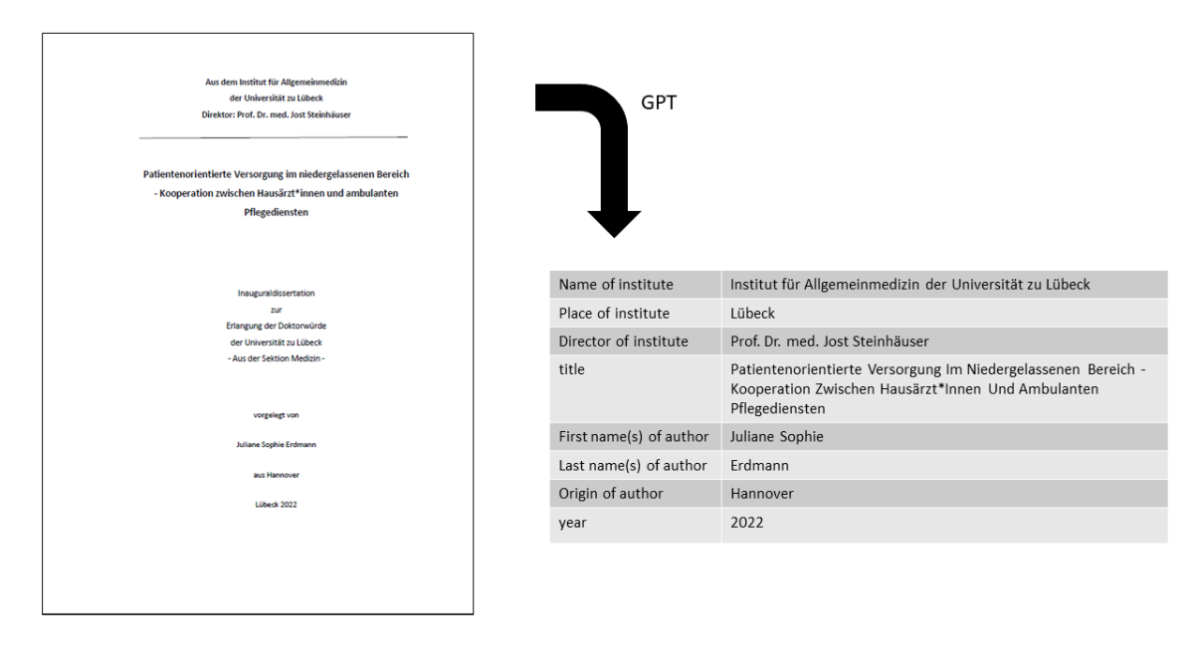
Automated extraction of dissertation metadata using GPT models. Example of structured metadata extraction from a dissertation title page (University of Lübeck, Institute of Family Medicine, 2022) using GPT models. The extracted fields include institute name, location, name of director, title, author information, and year of publication.

We defined the first page containing a 4-digit number as the title page. Using only the title page of each file, publication characteristics were extracted by using the OpenAI model “text-davinci-003”: name of the institution, city of the institution, director of the institution, first and last name(s) of the author, title of the dissertation, origin of the author, and year of publication (Figure 2). For each dissertation, J Cittadino cross-checked if the information extracted by the model was correct.

#### Gemini

In the Gemini pipeline, we used Google Cloud Platform for preprocessing and text generation. Using Workflows, Cloud Function, and Gemini-1.5-Flash with a temperature of 0.2, we designed the following pathway. First, DocumentAI was used to extract the text from all PDFs and store it in a JSON file [16]. Only using the first page and Gemini-1.5-Flash, we then extracted the same information from each PDF as with GPT-4o: name of the institution, city of the institution, director of the institution, first and last name(s) of the author, title of the dissertation, origin of the author, and year of publication.

#### Common Pathway

Next, for each LLM, we generated a string containing the whole text of the dissertation after the table of contents. The page number after the end of the table of contents was extracted by looping through snippets of the first 10 pages of each document using text-davinci-003 or Gemini-1.5-Flash.

Continuing the analytical pipeline, we searched the generated string for a summary section of the whole thesis. If found, the extracted paragraph was stored as *abstract_whole*. If not found, the text was scanned for paragraphs containing the subsections Aim of the study, Methods, Results, Discussion, and Conclusion as headings, which were extracted and summarized using text-davinci-003 or Gemini-1.5-Flash if found.

If a summary paragraph was identified, we shortened it to the first 4000 characters and used GPT-4o and Gemini-1.5-Flash to generate uniform abstracts containing the subheadings Aim of the study, Methods, Results, and Discussion. For both models, we used the same prompt and hyperparameters:

- Prompt: “Summarize the following summary section of a medical doctoral thesis into sections with the headings Objective, Methods, Results, and Discussion, each in no more than 2‐3 concise sentences: [summary section]” (German: “*Fasse die folgende Zusammenfassung einer medizinischen Doktorarbeit in Abschnitte mit den Überschriften Zielsetzung, Methodik, Ergebnisse und Diskussion knapp in jeweils maximal 2-3 Sätzen zusammen: [Zusammenfassung].*”
- Temperature: 0.2.
- Maximum number of tokens: 950.

The time needed for completion of the script as well as usage costs of the OpenAI LLMs were assessed.

#### Abstract Comparison

For each dissertation, researchers (PT, TS, J Cummerow, and LW) independently drafted an abstract and then agreed on a pooled version by discussion. The result was a third version of the abstract, this one human-generated. Afterward, the researchers estimated their time needed for drafting and agreeing on a pooled version. Then, three senior researchers (KG, KF, and CS), were asked to evaluate all three abstract versions in different orders, not knowing which two were LLM-generated and which one was human-generated. For each abstract version of each dissertation, we collected two ratings using German school marks between 1 (=best) and 6 (=worst). We then analyzed these ratings using descriptive statistics and a robust analysis procedure, the nonparametric Kruskal-Wallis test for independent groups [17]. Results were visualized in a boxplot.

#### Text Generation Evaluation

Words were counted for each generated abstract. To automatically evaluate the performance of LLM-generated abstracts, we used BERTScore (bidirectional encoder representations from transformers) [18] for contextual embeddings. This method uses the pretrained language model BERT to compare the similarity between tokens by calculating precision, recall, and the harmonic mean *F*_1_ [19]. All analysis processes were done using Python (version 3.10.1; Python Software Foundation) [20]. The code is available upon request.

#### Translation Evaluation

Finally, we used GPT-4o and Gemini-1.5-Flash to translate the newly generated abstracts into English. Following World Health Organization guidelines for translations [21] to make sure that the translation process did not result in the loss of information, we retranslated the generated English translation back into German again using the same LLM. Then, generated German translations were compared to the originally generated abstract (by J Cittadino).

## Results

### Overview

Paper-based documentation was more common (n=7) and often showed apparently poor digital quality after scanning. However, using the GPT-4o pipeline, we were able to extract text and information from all dissertations. Even so, we observed some common mistakes—for example, the German letter “ü” was often transformed to “ii.” Interestingly, while generating an abstract, both GPT models could still correctly understand the content and remove spelling mistakes.

### Thesis Characteristics

Information from the title page could be extracted for all theses, and the details were accurate except for two spelling mistakes (“é” instead of “ö”). There was no difference between OpenAI models and Google models. Dissertations covered various topics of family medicine research completed between 1965 and 2022 (Table 1).

**Table 1.** GPT-4o–generated characteristics of the included dissertations.

Name of institute	Institute location	Director of institute	Title of doctoral thesis	First name(s) of author	Last name(s) of author	Origin of author	Year(s)
*Institut für Medizinische Statistik der Freien Universität Berlin* [Institute of Medical Statistics, Free University of Berlin]	Berlin	Prof Dr med, Dr phil Karl Freudenberg	*Beziehungen zwischen Einweisungsdiagnosen und klinischen Diagnosen* [Relationships between admission diagnoses and clinical diagnoses]	Helmut	Pillau	Berlin	1965
*Universität Ulm (MNH)* [University of Ulm (MNH)]	Ulm	Priv Doz, Dr S Haussler	*Entwicklung kassenärztlicher Leistungen bei verschiedenen Arztgruppen im K. V. Bereich Nordwürttemberg (1965 - 1969)* [Development of services provided by contracted physicians in various medical groups in the North Württemberg Association of Statutory Health Insurance Physicians (1965 - 1969)]	Klaus	Besel	Vohringen/Iller	1965‐1969
*Institut für medizinische Statistik und Dokumentation* [Institute of Medical Statistics and Documentation]	Kiel	Prof Dr med G Griesser	*Analyse einer Allgemeinpraxis* [Analysis of a general practice]	Andreas	Kernbichler	Meldorf	1973
*Albert-Ludwigs-Universität Freiburg im Breisgau* [Albert Ludwigs University of Freiburg]	Freiburg im Breisgau	Honorarprofessor Dr med HH Schrömbgens	*Die Lehre der Allgemeinmedizin an den deutschen Hochschulen (1966 - 1978)* [Teaching of general practice at German universities (1966 - 1978)]	Claudia	Keller-Röll	Deggingen	1979
*Medizinische Poliklinik der Universität München* [Medical Outpatient Department, University of Munich]	München	Prof Dr N Zéllner	*Untersuchung zur Compliance bei der Diagnostik und Therapie des arteriellen Hypertonus in einer Hausarztpraxis in Stadtrandlage* [Study on compliance in the diagnosis and treatment of arterial hypertension in a suburban family practice]	Manfred	Lohnstein	Augsburg	1983
*Institut für Allgemeinmedizin* [Institute of General Practice]	Frankfurt AM Main	Prof Dr K Jork	*Selbstmedikation bei älteren Menschen und Studenten* [Self-medication among older adults and university students]	Elke	Iburg	Hannover	1991
*Philipps-Universität Marburg* [Philipps University of Marburg]	Marburg	Frau Prof Dr med Erika Baum	*Allgemeinmedizin in Großbritannien und Deutschland: Die Auswirkungen verschiedener Vergütungssysteme auf die Qualität präventiver Versorgung* [General practice in Great Britain and Germany: the effects of different remuneration systems on the quality of preventive care]	Norbert	Donner-Banzhoff	Viersen/Rheinland	1993
*Zentrum der Gesundheitswissenschaften Institut für Allgemeinmedizin* [Center for Health Sciences, Institute of General Practice]	Frankfurt AM Main	Prof Dr Ferdinand M Gerlach	*Möglichkeiten durch Delegation hausärztlicher Leistungen am Beispiel von Versorgungsassistentinnen in der Hausarztpraxis (Verah)* [Opportunities through delegation of family physician tasks using the example of practice assistants in family medicine (VERAH)]	Karola	Mergenthal	Büdingen, Hessen	2016
*Institut für Allgemeinmedizin der Universität zu Lübeck* [Institute of Family Medicine, University of Lübeck]	Lübeck	Prof Dr med Steinhäuser	*Nasa-Task Load Index - Ein Instrument, um sich der Komplexität von Beratungsanlässen in der Allgemeinmedizin zu nähern* [NASA Task Load Index: a tool for approaching the complexity of consultation situations in general practice]	Britta	Galler	Stade	2020
*Institut für Allgemeinmedizin der Universität zu Lübeck* [Institute of Family Medicine, University of Lübeck]	Lübeck	Prof Dr med Jost Steinhäuser	Patientenorientierte Versorgung im Niedergelassenen Bereich - Kooperation zwischen Hausärzt*Innen und ambulanten Pflegediensten [Patient oriented care in the outpatient sector: cooperation between family physicians and ambulatory nursing services]	Juliane Sophie	Erdmann	Hannover	2022

### Abstract Summary

Paragraphs containing a summary section of the whole dissertation could be found in 9 of 10 documents. In the case where no summary was found, a paragraph was extracted from the Discussion subsection and summarized using GPT-4o, while Gemini-1.5-Flash was able to provide a whole abstract. Thus, GPT-4o generated abstracts in 9 of 10 cases and Gemini-1.5-Flash in all 10 cases.

In the text extraction, we observed some common mistakes—for example, the German letter “ü” was often transformed to “ii.” The GPT model could still correctly understand the content and remove spelling mistakes while generating an abstract.

Running the script a second time resulted in the same extraction of all dissertation characteristics in 9 of 10 cases for GPT-4o and in all cases for Gemini-1.5-Flash. In one case, a different institute director was found, which was extracted as “Korefferent” and not “Referent.” Generated abstracts, however, differed in their wording while containing the same information.

Running the full script using the GPT-4o pipeline lasted 45.38 minutes (2720 s) and cost US $2.67. Additionally, we estimated a scanning time of 5-10 minutes for each dissertation. Each researcher spent about 1.5 hours drafting an abstract and about 0.5 hours while creating the pooled version.

### Abstract Comparison

**Figure 3.**
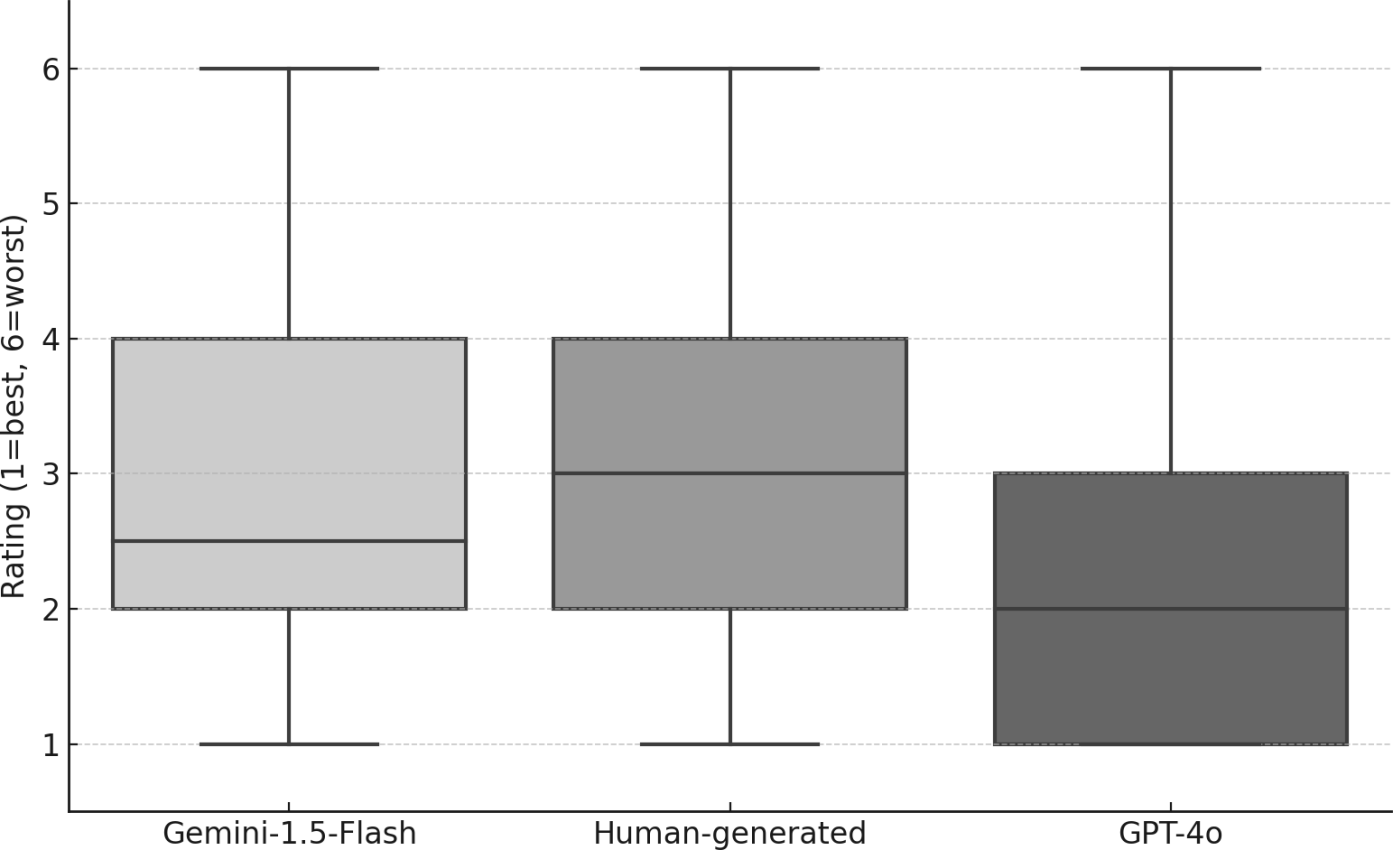
Distribution of abstract quality ratings across GPT models and human raters. Comparison of abstract quality ratings (1=best, 6=worst) generated by 3 sources (GPT-4o, Gemini-1.5-Flash, and human evaluators) for a sample of German medical dissertations. Boxplots show median, interquartile range, and variability of the assigned scores.

For each modality of abstract generation—GPT-4o, Gemini-1.5-Flash, or human-generated—two scores from different rater were collected. Scores covered the whole range from 1 to 6 for all modalities. Although there was no statistical difference in the nonparametric Kruskal-Wallis test for independent groups (*P*=.44), GPT-4o showed the best mean rating (2.44), while human-generated (3.00) and Gemini-1.5-Flash (3.25) were evaluated lower (Figure 3). Standard deviations (SD) were in the same range for all modalities (GPT-4o: 1.42; Gemini-1.5-Flash: 1.71; human-generated: 1.17).

### Text Generation Evaluation

LLM-generated abstracts consisted of fewer words compared to human-generated ones (mean word counts were 141 for GPT-4o, 137 for Gemini, and 352 for human-generated abstracts). Mean BERTScores showed moderate-to-high congruence between LLMs and human-generated abstracts (mean *F*_1_: GPT-4o: 0.72 and Gemini: 0.71, respectively; Table 2).

**Table 2.** BERTScore for the 10 dissertations for GPT-4o and Gemini.^a^

Dissertation ID	GPT-4o BERTScore	Gemini BERTScore
1	0.0	0.725
2	0.685	0.709
3	0.71	0.698
4	0.728	0.718
5	0.691	0.7
6	0.723	0.731
7	0.719	0.649
8	0.758	0.715
9	0.73	0.714
10	0.731	0.727
All	0.72	0.707

a BERT: bidirectional encoder representations from transformers.

### Translation Evaluation

When abstracts generated by both LLMs were translated into English and then back to German by using GPT-4o, the retranslated new German abstracts did not show a loss of information in comparison to the original German abstracts, although the wording was not exactly the same in all 10 cases.

## Discussion

### Main Findings

Results from this feasibility study suggest that LLMs can be used to extract relevant general characteristics from doctoral theses. Further, the models (GPT-4o and Gemini-1.5-Flash) accurately generated uniform abstracts 90% and 100% of the time, respectively. Machine-based translation of these abstracts into English did not show a loss of information. When asked, other researchers perceived these LLM-generated abstracts as similar to human-generated abstracts. BERTScores showed moderate-to-high similarity between LLM- versus human-generated abstracts. However, as hallucinations could not be totally eliminated, LLMs provide a tool to explore but not analyze medical dissertations.

### What Is Already Known

Medical knowledge is growing rapidly, and it is close to impossible to keep up with new insights and information [22,23]. Although health care systems currently use both paper-based and electronic health records [24], OCR can help to precisely extract text from scanned documents [13]. Lastly, accumulating evidence has showed that previously used rule-based or machine learning–based IE have been outperformed by deep neural networks even if pretrained, and they can help process a lot of data [25,26]. Although new, publicly available LLMs like ChatGPT and Gemini could possibly help in IE of medical documents, although ethical concerns have been mentioned [5,27]. Further, many projects have examined how and how well LLMs can help in IE of medical documents [28-32].

Although Recall-Oriented Understudy for Gisting Evaluation (ROUGE) [33] for overlapping unigrams is a known metric for the evaluation of text generation, we choose the BERTScore as our evaluation metric. BERTScore captures the contextual embedding and was thus more suitable to our study aim to examine how well LLMs can capture the content of long dissertations and generate an abstract including the relevant information [18]. The observed LLM performance in summarizing dissertations in our study was similar to reported *F*_1_ BERTScores in a Dutch study examining the differences in note-taking between humans and a digital scribe in a clinical workflow using various GPT models [34].

### What This Study Adds

To our knowledge, this is the first study examining the usage of GPT-4o and Gemini-1.5-Flash for IE in German doctoral theses. We actively chose a feasible method of digitization by using a standard multifunctional office printer, which is not in line with national standards of archiving documents, to simulate everyday conditions [9]. Our results suggest, however, that standardized IE through LLMs can be used to create uniform abstracts for dissertations that otherwise would remain difficult to access. Interestingly, these abstracts do not seem to be inferior to human-generated ones, indicated by the ratings of senior researchers. By costing as little as about US $0.25 and taking only 10-15 minutes (scanning: 5‐10 min, creation: 5 min) on average for each thesis, past results could be made more accessible and therefore easier to use for further research by using this analytical pipeline. This is especially true when considering that the creation of one pooled human-generated abstract took about 6 hours (360 min) and did not result in a higher rating from senior researchers, making it 24-36 times slower and more expensive.

A Dutch study examining note-taking performance reported that LLM-generated texts consisted of more words than human-generated texts (137 vs 101 words) [34]. However, we observed that human-generated abstracts were about 2.5 times longer than the ones from LLMs. This might be due to the need not to miss any important information when writing an abstract as a human and therefore generating a longer text. Further, LLMs might have difficulties capturing the whole extent of a dissertation, thus generating a shorter text. Two dissertations with low *F*_1_ BERTScores (IDs 2 and 5) were both written on typewriters, which might have resulted in informational loss during the OCR process, thus possibly accounting for a lower evaluation score. Conversely, we observed that dissertations written in Microsoft Word and converted to PDF achieved higher *F*_1_ BERTScores (IDs 8 and 10).

Hallucinations in medical LLMs persist [6], so users should be cautious about fully trusting the results. At this scientific stage, we propose using LLMs for medical dissertations as a tool for exploring—for example, for abstract generation—but recommend verifying generated information. By using this tool, we can unlock the rich data from doctoral theses in ADAM, making this valuable knowledge publicly accessible and strengthening up-to-date research.

### Strengths and Limitations

For this study, we only presented a small dataset including 10 doctoral theses of which the results can only be used for hypothesis generation and do not claim to be generalizable. Although we included files from 1965 and 2022, representing examples of the oldest and most recent documents in ADAM, further studies should include more dissertations to be more representative.

By using two recent LLMs (GPT-4o and Gemini-1.5-Flash), we could directly compare their respective results and performance. In this pilot study, however, we did not include additional models, which should be a focus of further studies. Additionally, because of the small sample size, we did not calculate interrater reliability among senior researchers, which might have biased ratings results. Although BERTScores have been effectively used in evaluating text generation by LLMs, they lack an understanding of nuances in medical language and therefore should be used with caution [35]. This underlines the need for an evaluation metric specifically to be used in medical contexts as potential threats from misinformation are among the most discussed risks of LLM usage [36].

### Conclusions

In this feasibility study using 10 medical dissertations completed between 1965 and 2022, we provide a proof of concept for the usage of LLMs, namely GPT-4o and Gemini-1.5-Flash, for IE from theses in ADAM. Within the constraints of a small, intentionally heterogeneous sample, results indicate promising feasibility. Using these models, typical publication characteristics as well as uniform abstracts could be generated, and they were not perceived differently to human-generated abstracts, while being about 30 times faster to generate.

However, as LLM hallucinations persist, research applications of LLMs should be approached with caution. Further research should include more dissertations, which will help researchers understand trends in various time periods and possibly allow for the grouping and summarizing of similar dissertations.

## Supplementary material

10.2196/77707 Multimedia Appendix 1 TRIPOD+LLM checklist.

## Abbreviations

ADAM: *Archiv der deutschsprachigen Allgemeinmedizi*n (English: Archive of German-language General Practice)
BERT: bidirectional encoder representations from transformers
DEGAM: German Society of General Practitioners and Family Physicians
IE: information extraction
LLM: large language model
OCR: optical character recognition
ROUGE: Recall-Oriented Understudy for Gisting Evaluation
TRIPOD: transparent reporting of a multivariable model for individual prognosis or diagnosis
